# A hybrid structure based on silk fibroin/PVA nanofibers and alginate/gum tragacanth hydrogel embedded with cardamom extract

**DOI:** 10.1038/s41598-024-63061-4

**Published:** 2024-06-18

**Authors:** Shadan Irantash, Adeleh Gholipour-Kanani, Najmeh Najmoddin, Mehdi Varsei

**Affiliations:** 1 Department of Biomedical Engineering, Science and Research Branch, Islamic Azad University, Tehran, Iran; 2 Department of Textile Engineering, Science and Research Branch, Islamic Azad University, Tehran, Iran; 3 Department of Biomedical Engineering, Medical Engineering and Biology Research Center, Science and Research Branch, Islamic Azad University, Tehran, Iran

**Keywords:** Hybrid structure, Silk fibroin nanofibers, Hydrogel, Tragacanth, Cardamom extract, Carbohydrates, Medical research, Biomaterials, Nanoscale materials

## Abstract

Hybrid structures made of natural-synthetic polymers have been interested due to high biological features combining promising physical–mechanical properties. In this research, a hybrid dressing consisting of a silk fibroin (SF)/polyvinyl alcohol (PVA) nanofibers and sodium alginate (SA)/gum tragacanth (GT) hydrogel incorporating cardamom extract as an antibacterial agent was prepared. Accordingly, SF was extracted from cocoons followed by electrospinning in blend form with PVA (SF/PVA ratio: 1:1) under the voltage of 18 kV and the distances of 15 cm. The SEM images confirmed the formation of uniform, bead free fibers with the average diameter of 199 ± 28 nm. FTIR and XRD results revealed the successful extraction of SF and preparation of mixed fibrous mats. Next, cardamom oil extract-loaded SA/GT hydrogel was prepared and the nanofibrous structure was placed on the surface of hydrogel. SEM analysis depicted the uniform morphology of hybrid structure with desirable matching between two layers. TGA analysis showed desired thermal stability. The swelling ratio was found to be 1251% after 24 h for the hybrid structure and the drug was released without any initial burst. MTT assay and cell attachment results showed favorable biocompatibility and cell proliferation on samples containing extract, and antibacterial activity values of 85.35% against *S. aureus* and 75% against *E. coli* were obtained as well. The results showed that the engineered hybrid nanofibrous-hydrogel film structure incorporating cardamom oil extract could be a promising candidate for wound healing applications and skin tissue engineering.

## Introduction

Skin is the first line of defense of the body which protects it against environmental dangers and trauma such as wounds, burns, surgery and radiation^1^. In order to address the serious issues caused by traditional and common methods of wound healing such as infection or leaving scars, novel wound dressing systems have been introduced, which have shown success both in research studies and in market^2^. Nevertheless, ideal wound dressings need to meet different requirements for use in clinical settings^3^. They should be biocompatible and hydrophilic, have hemostatic properties, prevent infection, relieve pain by providing a cool feeling for the patient, absorb exudate to minimize bacterial activity, form a barrier against the environment, not adhere to the newly forming skin^4^, possess favorable gas and vapor permeation and accelerate the skin regeneration^5^. Contrary to popular belief, it has been shown that a moist environment can accelerate healing and prevent scarring through autolytic debridement, reduce pain, activate collagen synthesis, aid the migration of keratinocyte over the wound surface and support the transfer of nutrients, growth factors, and other soluble materials necessary for skin formation in the wound site. Hence, wound dressing materials with favorable water retention properties can facilitate the healing process^6^.

Wound dressings can be prepared in various forms such as hydrogels, sponges, mats, membranes, films and colloids^7,8^. Among them, electrospun dressings have high porosities, controllable degradability profiles, and favorable permeation properties^9^. The porosity and large surface area of electrospun mats enable the absorption of exudates from the wound and also permeation of vapor from the surface of the dressing^10^ They also can form the protective barrier for the susceptible tissue against environmental factors^11^. On the other hand, hydrogels are 3D uniform structures with high water retention capability based on physically- or chemically-crosslinked polymers. Their structures mimic the extracellular matrix of tissues, and they can provide a comforting feeling for patients through the absorption of exudates. Moreover, hydrogels alleviates pain through protecting exposed peripheral nerve terminals^12,13^.

Wound dressings can be prepared based on natural or synthetic materials or combination of both that can profit from properties of both types of materials^14^. Natural polymers are sustainable and environmentally friendly, and induce less reactions in the immune system due to the similarity to the tissues in the body^15^; Natural polymers such as chitin, chitosan, gelatin, dextran, cellulose, silk, starch, alginate, different gums, and their derivatives have been reported for wound healing^16^. Alginate is a linear polysaccharide derived naturally from algae or bacteria^17^. This natural polymer is hydrophilic and biocompatible with high liquid-absorbing capacity. Sodium alginate (SA), which is the sodium salt of alginic acid, can absorb moisture double its own weight, which compared to common gauze is 5–7 times more, making it an interesting choice for wound dressing applications^18^. It has been reported that re-epithelialization as a vital step in the complex process of wound healing could be accelerated in the presence of Sodium alginate. In addition, faster angiogenesis, granulation tissue formation and wound closure can be induced by using systems based on SA^19^. Gum tragacanth (GT) is a hypoallergenic, non-toxic and hydrophilic natural anionic polysaccharide for biomedical applications^20^. GT has been studied in the form of topical creams, hydrogels and composites for wound healing, and has been shown to induce wound contraction and healing^21^. Recently, it has been reported that the tragacanth gum (TG) has inherent wound healing potential which fasten the wound healing. Tragacanth is also effective in the proliferation and remodeling phases of wound healing^21^. Its antioxidant activity helps in wound healing processes, In addition to being cost-effective, easily accessible and sustainable, GT can increase mechanical properties, water uptake and degradation rate^22^.

Silk has been used from ancient times in medicine and its derivatives such as silk fibroin (SF) have been used in wound dressings owing to its biocompatibility and biodegradability with favorable mechanical properties, water absorption capacity and low immunogenicity^23^. The raw silk is a composite fiber composed of fibroin and sericin, which are proteinaceous materials. Sericin is a water soluble glue, which can be named as gum or natural sizing agent. As the name implies, it bonds fibroins together. In addition, SF has been shown to stimulate cell migration and proliferation, which can improve skin regeneration. SF can also control the release of pharmaceuticals, leading to formation of systems with sustained release^24^.

While natural polymers possess excellent features for wound dressing application, their high surface tension and viscosity make challenges during electrospinning process. Furthermore, they suffer from unfavorable mechanical properties. To overcome such drawbacks, synthetic polymers including polyurethane, poly-ɛ-caprolactone, polyvinyl alcohol (PVA), cellulose acetate, polyglycolic acid, polylactic acid, polyacrylic acid, and polyethylene glycol could be utilized along with natural ones for wound dressing^25,26^. One of the most commonly used polymers in skin tissue engineering based on the electrospinning method is PVA. PVA is a synthetic cost-efficient polymer with favorable water-solubility, nontoxicity, and biodegradability which has been widely used in wound dressing applications^27,28^. Since PVA can be produced in a wide range of molecular weights, it can be used to control the viscosity of electrospinning solution, tailor the properties of resulting fibers, and aid the spinning process for natural polymers that cannot be electrospun alone^27^.

Recently, plant-based antibiotic agents as an alternative for synthetic antibiotic agents have been investigated to prevent infection in the wound site and also decrease the dangers of antibiotic resistance as well as exploiting natural and sustainable recourses^29^. Cardamom (*Elettaria cardamomum*) extract constitutes various polyphenolic acids and flavonoids with the ability of disrupting bacteria membrane which gives it antibacterial properties^30^. Moreover, studies show that ethanolic extract of Cardamom has antibacterial activity against multi drug resistant bacterial strains, showing the potential of this plant as safe and effective substitute of synthetic antibiotics with far less side effects^31^. In addition to anti-oxidant activity, due to the inhibition of the nuclear factor kappa-B (NF-κB) signaling pathway, which is one of the inflammatory signaling pathways, it also has possess anti-inflammatory properties, making it a great candidate for utilization as an agent that can manage wound infection and induce skin regeneration in wound dressing systems^32^.

Hence, in this study, a hybrid wound dressing system that took advantage of both electrospinning process and hydrogels was prepared. SF was electrospun with the aid of PVA, and was covered on a composite hydrogel comprising cardamom oil extract based on GT and SA. Prepared hybrid hydrogels underwent different physicochemical and cell studies to investigate their efficiency for skin tissue engineering and wound healing applications.

## Materials and methods

### Materials

Silk (*Bombyx mori*) cocoons and gum tragacanth (GT) were purchased from Attarak Company (Iran). Cardamom was purchased as an oil extract from Giah Taghdis Company (Iran). Sodium alginate (SA) and glutaraldehyde (GA) were purchased from Sigma-Aldrich (Germany). Polyvinyl alcohol (PVA) (M_w_ = 72,000 g mol^−1^), calcium chloride dehydrate, lithium bromide (LiBr) and sodium carbonate (Na_2_CO_3_) were purchased from Merck (USA).

### Extraction of silk fibroin

**Figure 1:**
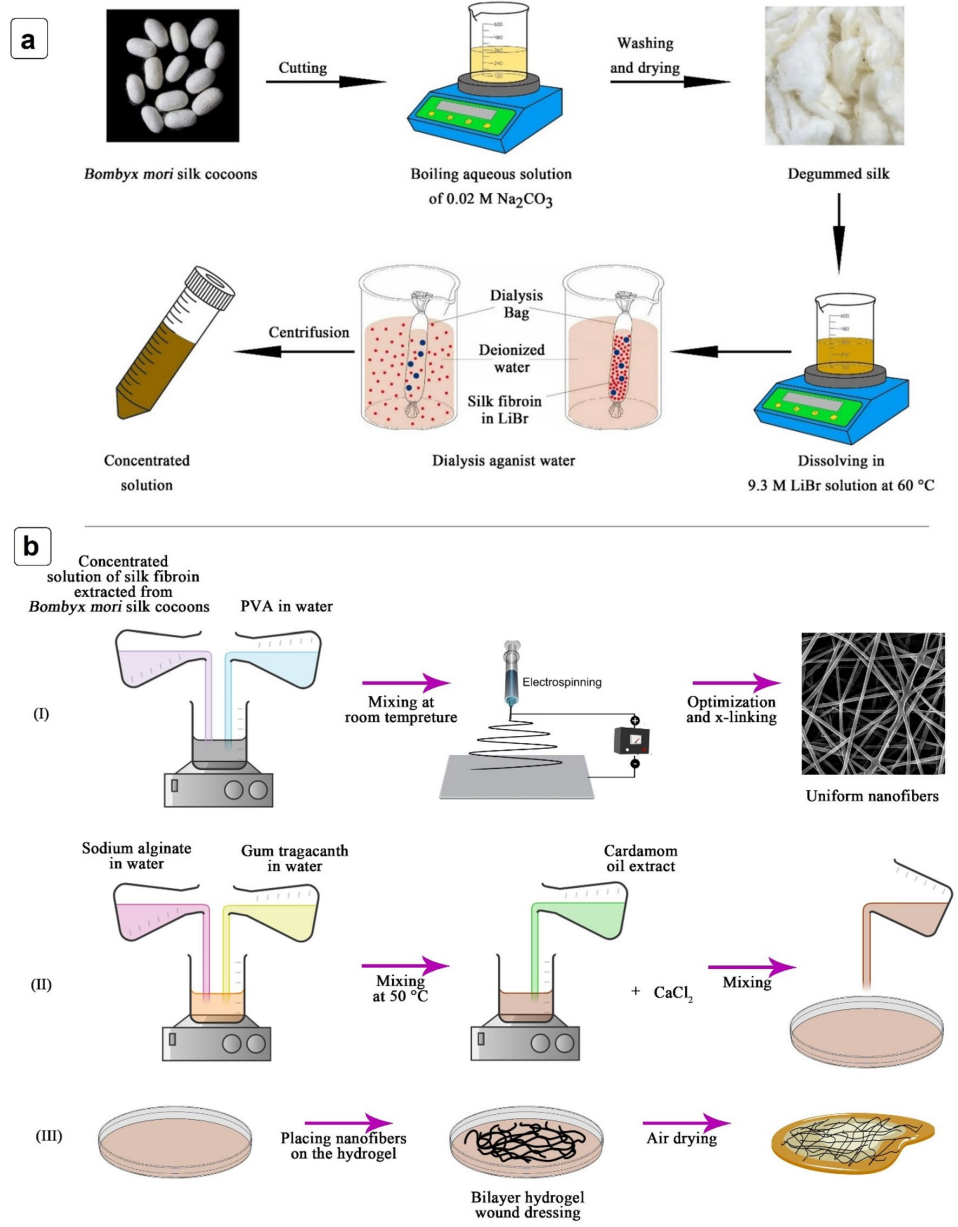
(**a**) Steps of extracting silk fibroin (SF) from cocoons; (**b**) the illustration of preparing hybrid structure.

The method for silk fibroin extraction was performed in accordance with the relevant guidelines and regulations reported by Cao et al.^33^. Briefly, 5 g dried *Bombyx mori* silk cocoons were cut into small pieces and stirred in a boiling aqueous solution of 0.02 M Na_2_CO_3_ for 20 min. The whole mass was washed with cold distilled water 3 times and then dried overnight. The solution of extracted SF was prepared by dissolving degummed silk in a 9.3 M LiBr solution at 60 °C for 4 h. The solution was dialyzed in a dialysis bag against deionized water for 3 days and the water was refreshed every 6 h in order to remove LiBr. After carrying out the dialysis step, a concentrated solution was obtained by centrifuging the SF solution at 4 °C and 9000 rpm for 20 min. The concentrated solution was stored at 4 °C for further studies. The process is shown in Fig. 1a.

### Preparation of SF/PVA nanofibers

After preparing a solution of 10% (w/v) PVA in distilled water, SF solution was added to the PVA and SF/PVA solutions with 1:1, 2:1 and 3:1 (v/v) ratios were obtained. The classical electrospinning approach (FNM Duos Electroris, HV35P OV, Fanavaran Nanomeghyas Co., Iran) was used to fabricate the fibers under the spinning condition of 15 kV and 18 kV applied high voltage, 10 cm and 15 cm nozzle to collector distance, and a flow rate of 0.5 ml/h. The nanofibers were collected on aluminum foil (Fig. 1b). Optimum electrospinning conditions were obtained through morphological analysis.

In order to enhance integrity and reduce electro spun fibers solubility in water, chemical crosslinking was performed in the vicinity of GA vapor. Accordingly, 30 ml GA was poured in a desiccator and electrospun nanofibrous samples were placed in it for 18 h at room temperature. Next, crosslinked samples were washed 3 times using phosphate-buffered saline (PBS) solution in order to remove unreacted residues and ensure insolubility in aqueous solutions.

## Preparation of the hybrid structure of nanofibrous mat-hydrogel film

2 wt% solutions of GT and SA were first prepared separately by dissolution in distilled water under constant stirring. SA and GT solutions were then mixed together (1:1) and stirred for 1 h at 50 °C. Next, cardamom oil extract (20 wt%) was added to the mixture and after mixing for 15 min, CaCl_2_ (1.5% w/w) was added as a crosslinking agent and the solution was mixed for another 30 min. The solution was poured in a petri dish (thickness = 3 mm). The petri dish was placed in a vacuum oven for 1 h at 50 °C. Next, the crosslinked electrospun nanofibers were placed on the surface of the hydrogel and left to dry at room temperature overnight. The schematic illustration of the process is shown in Fig. 1b.

### Morphological and physico-chemical characterization

Scanning electron microscopy (SEM) was used to investigate the morphology of the electro spun nanofibers and hybrid hydrogels using a JEOL SEM device (JSM5300, JEOL Ltd., Tokyo, Japan) after gold-sputtering of dried samples. Average fiber diameters were then measured by Digimizer software (v. 6.3.0) from SEM images. X-ray diffraction (XRD) patterns were recorded in the Braggs angle 2θ range of 10°–80° at room temperature. Accordingly, radiation was produced from a Cu anode tube (λ = 1.5405 A°) utilizing a Philips PW1730 X-ray generator (Netherlands) that worked at 40 kV, 30 mA, and at a scanning rate of 0.02°/s. Fourier Transform Infrared (FTIR) spectroscopy was performed to study the chemical bonds of the scaffolds and investigate the interactions between components in the samples using potassium bromide disks in the range of 4000–400 cm^−1^ by a EQUINOX 55 spectrophotometer (Bruker, Germany). Thermal properties were investigated by thermo gravimetric analysis (TGA) using a Q600 model from TA (USA) in the 35–500 °C range in air atmosphere.

Tensile strength was performed using a universal testing machine (Instron 5567) at room temperature in order to evaluate the mechanical properties of the hybrid structure according to the ISIR 2794 method. The rectangular samples with dimensions of 3 cm × 1 cm were mounted on the device in a paper frame with dimensions of 3 cm × 3 cm. The average thickness of layers was measured with a digital micrometer (Mitutoyo, Tokyo, Japan). The average thickness of nanofibrous layer, hydrogel layer and the hybrid structure was respectively about 50 µm, 150 µm and 170 µm. The tensile strength test was done at a strain rate of 50 mm/min. The samples are subjected to tension from both sides in line with the direction of the axis of load application. The test was repeated three times.

### Swelling studies

Square samples with 1 cm^2^ dimensions were cut and dried at 60 °C until constant weight (*W*_*d*_) was obtained. Then, they were immersed into 10 ml water at 37 °C with pH of 7.4 for 24 h. Next, the samples were removed from the solution and their surface was dried and they were weighted (*W*_*s*_). Here, the swelling ratio (DS) was defined using the following Eq. (1):

1 $$\% DS = \left[ {\frac{{W_{s} - W_{d} }}{{W_{d} }}} \right] \times 100$$

where, *W*_*d*_ and *W*_*s*_ were the weights of dried and swollen samples, respectively.

### Drug release studies

50 mg of the hybrid samples comprising cardamom oil extract were immersed in 100 µl of PBS buffer and placed in a 37 °C incubator and measurements were carried out at predetermined time intervals. The optical absorption of the samples was read at a wavelength of 240 nm using a NanoDrop UV–Vis spectrometer from Thermo Fisher (USA).

### Antibacterial activity

*Escherichia coli* (*E. coli*, ATCC 25922) and *Staphylococcus aureus* (*S. aureus*, ATCC 6538) bacteria were purchased from Pasteur Institute of Iran (Tehran, Iran) to study the antibacterial properties of hybrid hydrogel sample without extract, and the sample with cardamom oil extract. All bacterial strains were stored in a medium containing 11 v/v% dimethyl sulfoxide at – 70 °C before use. In order to perform the analysis, the bacterial strains were grown on Mueller Hinton Agar medium plates (Merck, Germany) at 37 °C. Single colonies of bacterial samples were cultured linearly in N-agar medium plates (NB 8g + agar 16 g) and placed in an incubator at 37 °C for 24 h. After two successive cultivations, the samples were rejuvenated and prepared for the next steps. The inoculum contains a suspension of microorganisms in the corresponding liquid culture medium with about 10^6^ colony forming units (CFU). To prepare the cell suspension, pure bacterial colonies were added to 10 ml of NB culture medium and after 30 s of vortexing, they were placed on a shaker-incubator for 4 h. The concentration of the samples in this time period was equal to the turbidity of half McFarland visually or equivalent to 0.5–0.6 in optical absorption of 600 nm and pure colonies were used to prepare the inoculation liquid (approximately 1.5 × 10^6^ CFU/ml). 100 µl incubated bacteria liquid was cultured on the sterilized Mueller Hinton Agar medium plates using sterilized swap. Gentamycin antibiotic papers (6 mm) were used as a control. Hydrogel samples were cut into disks (∼1 cm in diameter), placed on plates after placing one drop of fibroin for 15 min on them, and plates were placed in an incubator at 37 °C. Inhibition zones were investigated after 18 h.

### Cell cytotoxicity and cell attachment

In this study, mouse fibroblast L929 cells (NCBI C161) were purchased from Pasteur cell bank (Tehran, Iran) and used for cell viability studies. To investigate the cell proliferation rate, first 1 $\times$ 10^4^ cells along with 100 µl of culture medium (Dulbecco's Modified Eagle Medium DMEM containing 10 µl of FBS) were poured into each well of a 96-well cell culture plate and then placed in an incubator at 37 °C for 24 h until the cells adhere to the bottom of the plate. Next, the culture medium on the cells was removed as much as possible and 90 µl of the extract obtained from the samples along with 10 µl of fetal bovine serum (FBS), were added to each culture well and the cells were kept in the vicinity of these concentrations for another 24 h. After that, the culture medium was removed and 100 µl of (3-[4,5-dimethylthiazol-2-yl]-2,5 diphenyl tetrazolium bromide) (MTT), with a concentration of 0.5 mg/ml was poured into each well and placed in an incubator for 4 h. After 4 h, the solution was removed from the cells and isopropanol was added to dissolve the purple crystals. To better dissolve the MTT sediment, the plate was placed on the shaker for 15 min. Then, the concentration of the substance dissolved in isopropanol was calculated using an Eliza reader device (BioTek ELx808, USA) at a wavelength of 570 nm. The well with more cells shows a higher optical density (OD) than the well with fewer cells. Therefore, it could be determined from the relationship below the well with more cells and compared with the control sample. It should be noted that each sample had 5 repetitions and the cell viability percentage was computed using Eq. (2).

2 $$Viability\,\% = \frac{{mean\;{\text{OD}}\;{\text{of}}\;{\text{sample}}}}{{mean\;{\text{OD}}\;{\text{of}}\;{\text{control}}}} \times 100$$

Attachment of cells on scaffolds was also investigated after 1, 2 and 3 days by chemical fixation of cells (incubation with *P. fluorescens* and gently washing with pre-warmed medium to remove unattached cells), followed by a series of dehydration and drying procedures as described by Fratesi et al.^34^.

### Statistical analysis

In this study, data were represented as mean of replicates ± standard deviations (SD). One-way ANOVA followed by Tukey post *hoc* analysis was applied for statistical comparing of the means in different groups. Whole statistical computations were carried out using SPSS software (v. 16.0). Differences were considered to be statistically significant when the *P* value was lower than 0.05 (*P* < 0.05).

## Results and discussion

### Characterization of extracted SF

**Figure 2:**
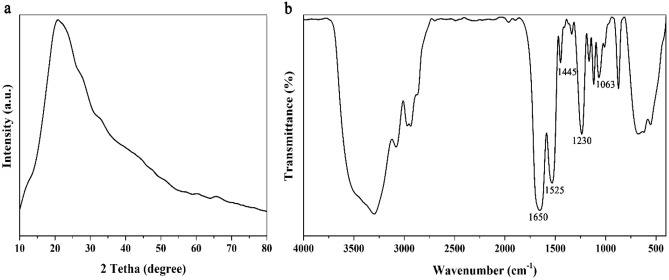
(**a**) XRD pattern and (**b**) FTIR spectrum of extracted silk fibroin (SF).

Extracted SF was characterized using XRD and FTIR analysis before further use. Accordingly, the XRD pattern (Fig. 2a) showed that the structure of SF was predominantly amorphous. The eminent single peak observed at 2θ = 20.1° was an indication of the silk II crystalline structure, similar to previous reports^35^.

The FTIR spectrum resulted from analyzing the chemical structure of extracted SF is shown in Fig. 2b. Accordingly, the peaks observed at 1063, 1230, and 1445 cm^−1^ could be ascribed to the stretching vibrations of C–O–C bonds, stretching vibrations of C–N bonds, and bending vibrations of C–H of SF, respectively. Moreover, peaks located at 1525 and 1650 cm^−1^ were related to bending vibrations of N–H groups and bending vibrations of O–H groups, respectively. Peaks observed in the 3000–3600 cm^−1^ range were related to stretching vibrations of N–H and O–H bonds. The results were in line with previous work reported in literatures^36–38^.

### Morphological analysis

#### Morphological study on nanofibrous layer

Similar to many natural biopolymers, SF cannot be easily electrospun alone and thus is commonly mixed with other natural or synthetic polymers for electrospinning^39^. SF has a negative charge on its backbone and when it is exposed to an electrical field, similarly-charged chains cause repulsion and hence prevent the success of electrospinning. PVA acts as a lubricant and neutralizes the repulsive forces of negative charges by slipping between SF chains. Thus, uniform electrospun SF-PVA fibers could be obtained^25^.

**Figure 3:**
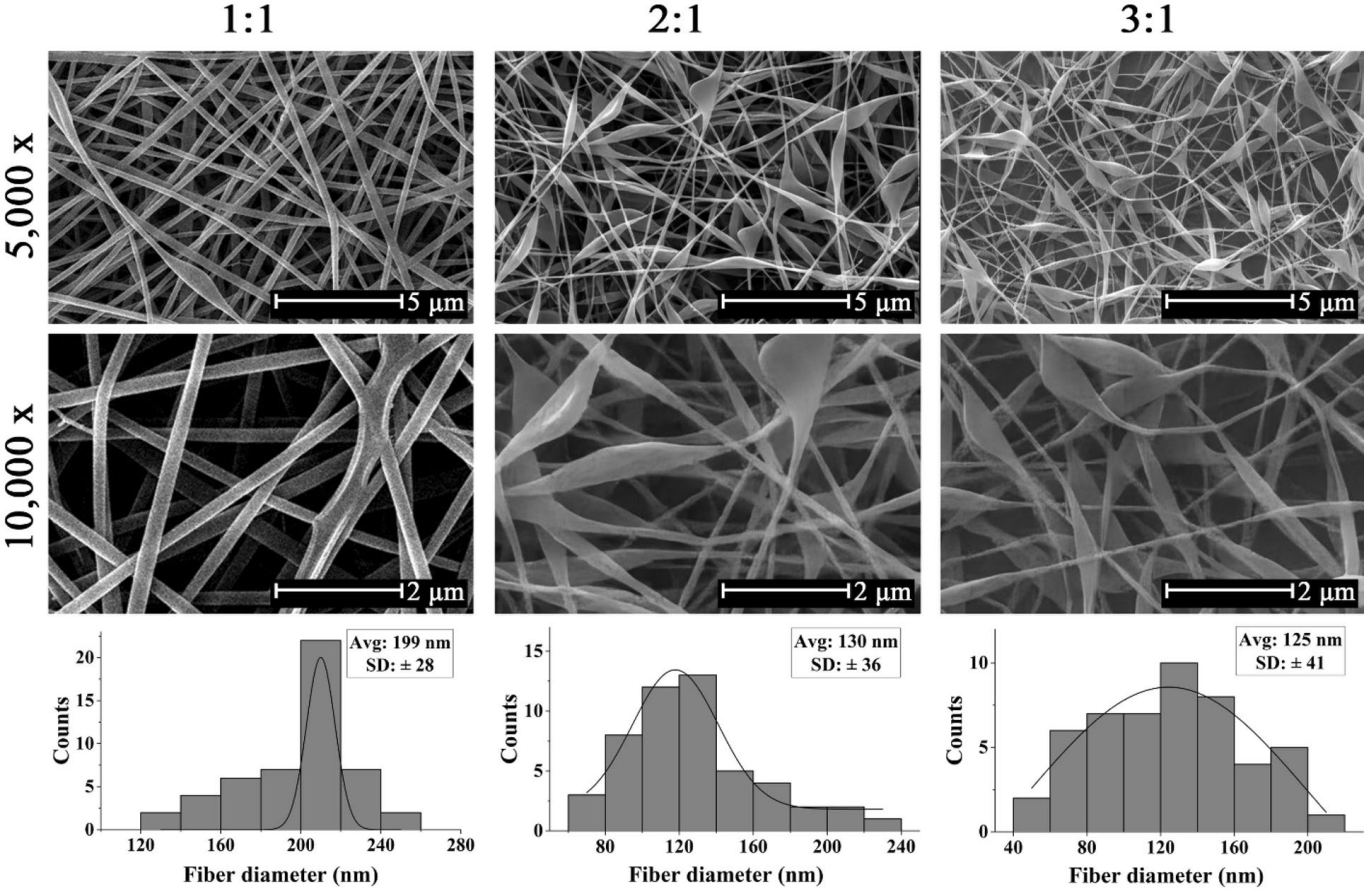
SEM micrographs and distribution charts of SF/PVA nanofibers with 1:1, 2:1, and 3:1 ratios electrospun at 18 kV and 15 cm nozzle to collector distance (magnifications = 5000 × and 10,000 ×).

In order to investigate the effect of the mass ratio of polymer contents on the morphology of fibers, SF/PVA nanofibers were electrospun at 1:1, 2:1 and 3:1 ratio at the constant electric voltage of 18 kV and nozzle to collector distance of 15 cm and resulting mats were investigated using SEM in Fig. 3. As shown in Fig. 3, the SF/PVA nanofibers with 1:1 ratio were uniform, smooth and bead-less with random orientation. Increasing the PVA content (in other words, lower SF content in the solution) increased the total concentration from 8.5% for 3:1 solution to 8.7% and 9% for 2:1 and 1:1 solutions, which increased the viscosity. Hence, it was suggested that for the 1:1 solution, PVA polymer chains slipped between SF chains more uniformly and could lead to more uniform fiber diameters by neutralizing electrical charges and hence creating a stable jet that could be formed more readily under the force of the electric field.

While for the sample with 1:1 ratio an average fiber diameter of 199 ± 28 nm was obtained, for samples with 2:1 and 3:1 SF/PVA ratios, the average fiber diameter values decreased, resulting in 130 ± 36 and 125 ± 41 nm diameters due to the lower concentration and viscosity of the solutions. This could be due to the fact that decrement of solution concentration followed by reduction of viscosity, diminished the chance of the formation of a stable jet on the nozzle and hence led to the formation of fibers that had structural defects. Hence, the scaffold resulted from the 1:1 SF/PVA solution was chosen as the optimum sample.

**Figure 4:**
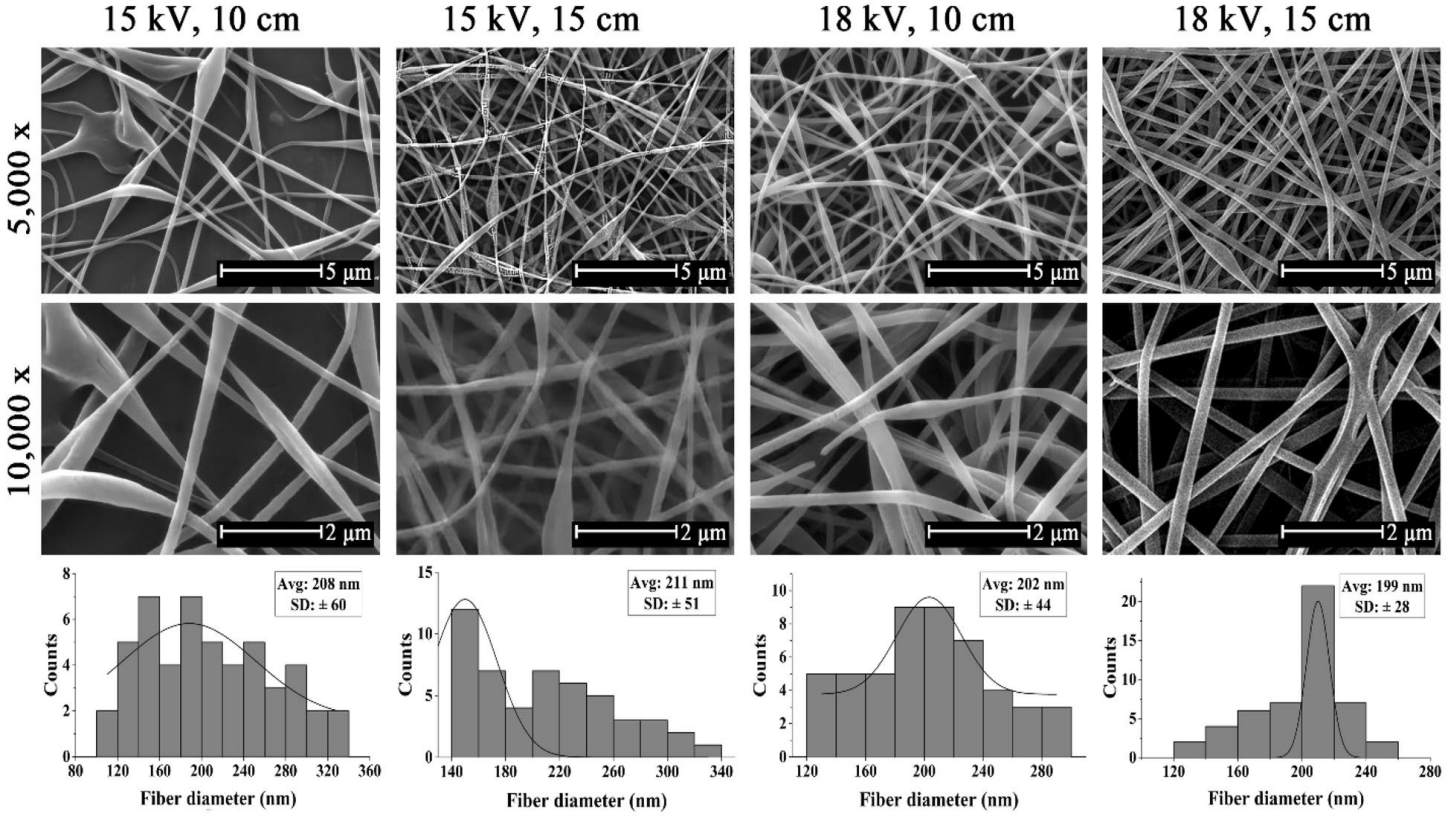
SEM micrographs and distribution charts of 1:1 SF/PVA nanofibers electrospun at 15 and 18 kV and 10 and 15 cm nozzle to collector distance (magnifications = 5000 × and 10,000 ×).

In order to determine the optimum electrospinning conditions, SF/PVA solution with 1:1 ratio was electrospun at 15 kV and 18 kV electrical voltages and at nozzle to collector distance of 10 and 15 cm and results is shown in Fig. 4. At the electrospinning condition of 15 kV applied voltage and 10 cm distance, beads were observed in the fibrous structure, and a high variation in fiber diameter was observed. It seems that increasing electrical voltage and nozzle to collector distance had a positive effect to obtain uniform and bead-free structure. Additionally, the average electrospun fibers diameters at voltages of 15 kV and 18 kV were found to be 206 ± 60 nm and 202 ± 44 nm for nozzle to collector distance of 10 cm and 211 ± 51 and 199 ± 28 nm for nozzle to collector distance of 15 cm, respectively. At a constant nozzle to collector distance, lower average fiber diameters were obtained in a stronger electrical field due to higher tension of the field. Additionally, increasing the nozzle to collector distance forced the solution to undergo higher stretching, which caused lower average fiber diameters at a constant voltage. The result is in line with the study reported by Fathi et al.^39^.

To conclude, the optimum electrospun conditions were obtained at a nozzle to collector distance of 15 cm and electrospinning voltage of 18 kV for the SF/PVA solution with 1:1 ratio, which resulted in the average fiber diameter of 199 ± 28 nm with uniform and bead-free structure.

#### Morphological study on hybrid structures

**Figure 5:**
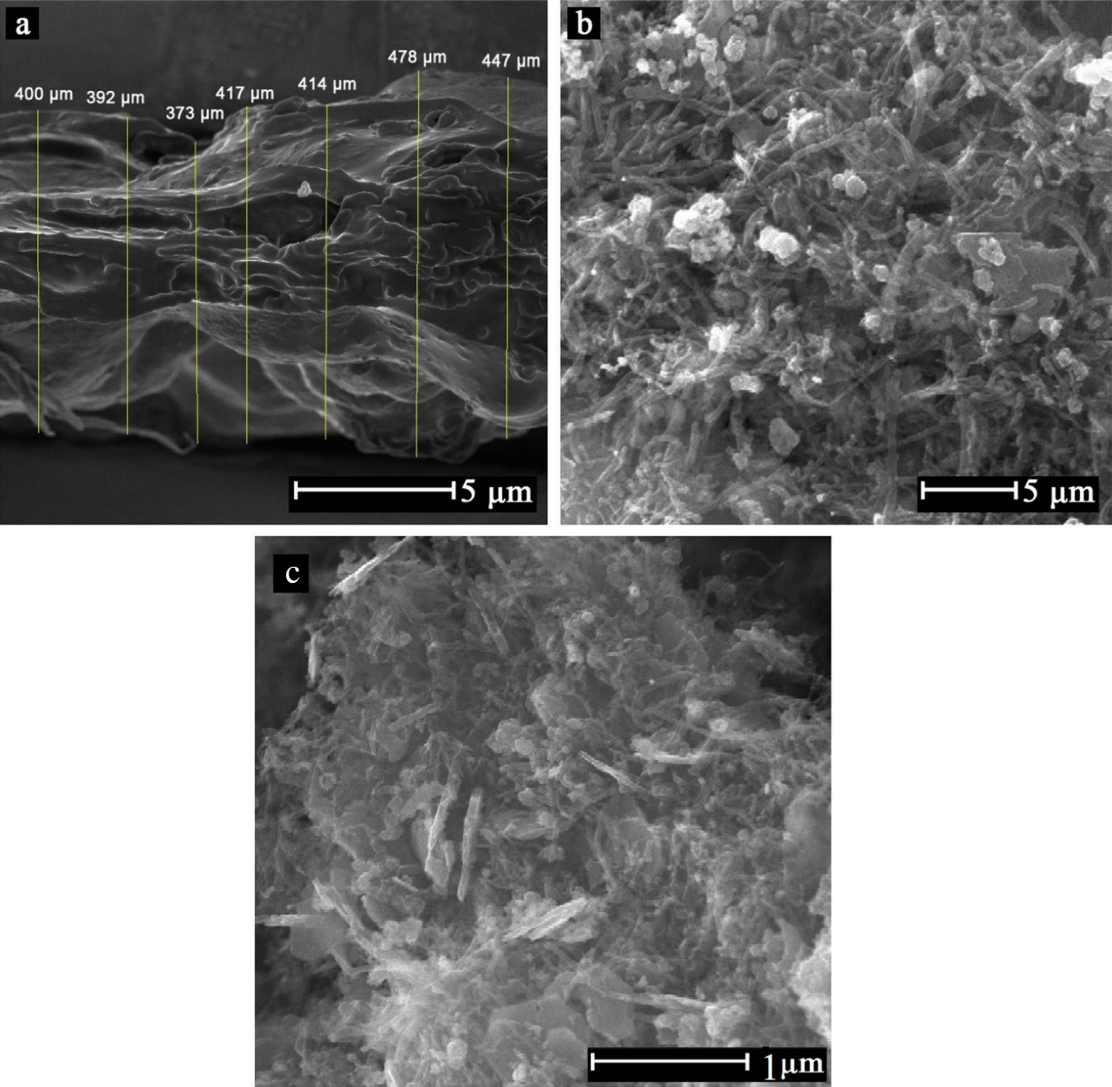
SEM micrographs of hybrid hydrogels demonstrating (**a**) the cross-section and (**b**, **c**) the surface of hybrid structure in different magnifications.

SEM analysis was also used to investigate the morphology of hybrid hydrogel based on SA/GT incorporated with SF/PVA nanofibrous layer, as shown in Fig. 5. Accordingly, the SF/PVA nanofibrous layer was observed on the surface of the SA/GT blend hydrogel. Not only did the hydrogel encompass the nanofibrous layer, but also penetrated into the fibrous structure, which could enhance the properties of the hybrid wound dressing.

### Crystalline and chemical structure of SF/PVA nanofibers and the hybrid structure

**Figure 6:**
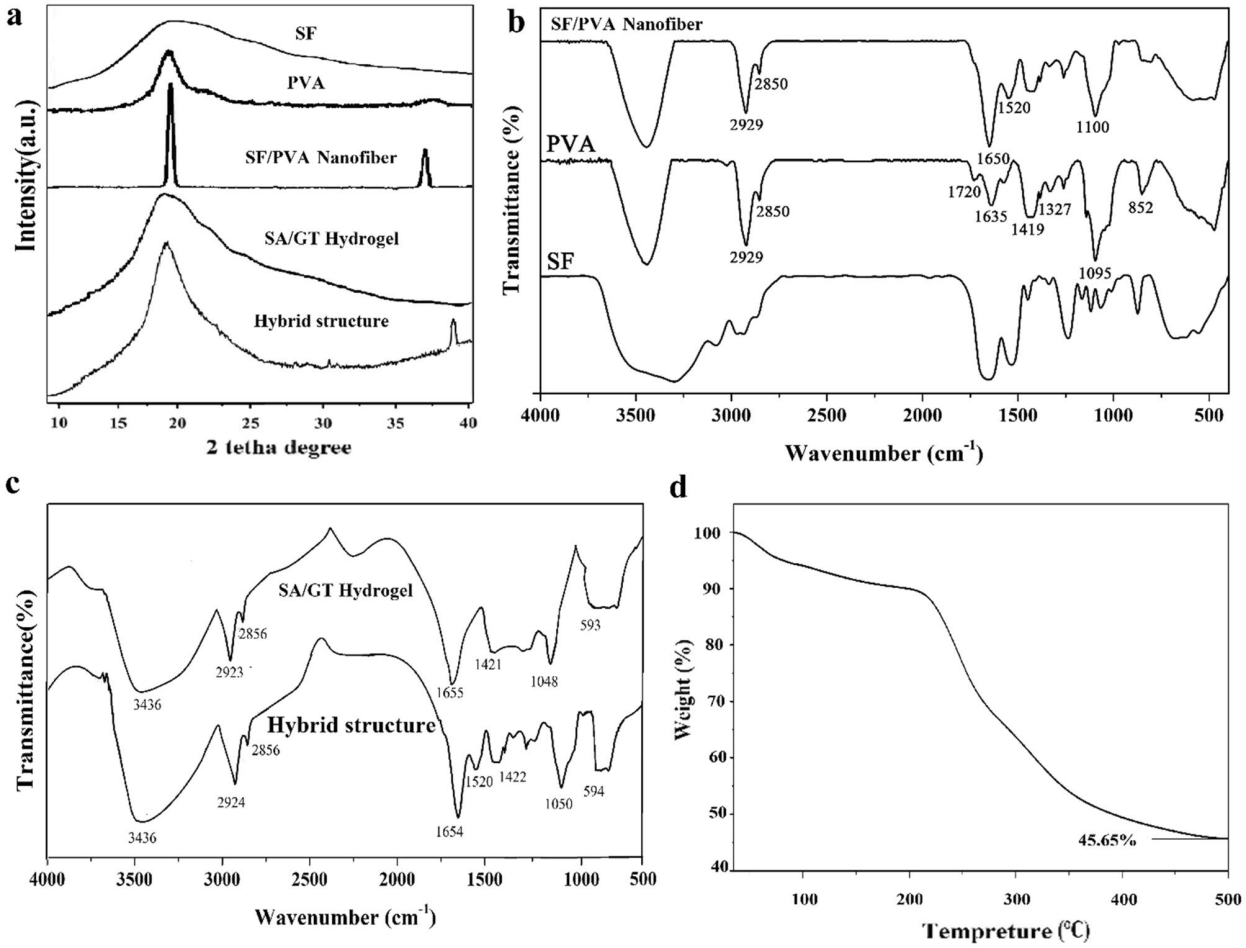
(**a**) XRD patterns, (**b**) FTIR spectra of SF, PVA, and SF/PVA samples, (**c**) FTIR spectra of sodium alginate/tragacanth (SA/GT) hydrogel and the hybrid structure of SF/PVA nanofibers- SA/GT hydrogel and (**d**) results of TGA experiment for hybrid hydrogel.

XRD analysis was used to evaluate the crystalline structure of SF, PVA, and their mixture, as shown in Fig. 6a. For pure PVA, two peaks at 2θ = 19.1°, 40.9° were observed which could be related to the semi-crystalline nature of PVA. On the other hand, SF/PVA sample had a peak at 19.8° corresponding to the β-crystalline lattice in addition to the peak at 19.9°, which was attributed to the apparent superposition of both materials. In the SF/PVA mixture, these peaks were attributed to the β-sheet silk II structure. Silk II is the pleated form of silk comprising of β-form or anti-parallel crystals^40^. Results showed that the crystalline structure of SF was affected by the PVA in the mixture, and shorter SF chains could initiate the crystallization of PVA, resulting in more distinctive lattices. The XRD graph of Alginate/GT showed a peak at 2θ = 20° of high intensity which indicated an amorphous structure of hydrogel. These results have also been observed in previous reports^41^.

Chemical interactions between the components of nanofibers were analyzed using FTIR spectroscopy and results are shown in Fig. 6b. For the PVA sample, the peak observed at 852 cm^−1^ was related to the rocking vibrations of C–H bond of PVA. Also, peaks observed at 1327, 1419, and 1635 cm^−1^ were related to bending and stretching vibrations of C–H of methyl and methylene bonds, and the bending vibrations of O–H of PVA, respectively^40^. Peaks observed at 1095 and 1720 cm^−1^ were both related to the C–O stretching bonds, respectively; and peaks observed at 2850 and 2929 cm^−1^ were related to the symmetric and asymmetric stretching vibrations of the C–H bond. In addition, peaks in the 3300–3600 cm^−1^ range were related to the stretching vibrations of O–H bonds due to the presence of hydroxyl groups^42^.

In FTIR spectrum of SF/PVA sample, peaks observed at 1230, 1520, and 1650 cm^−1^, were related to the stretching vibrations of C–N bonds, bending vibrations of N–H, and the bending vibrations of O–H in SF, as previously reported by others^40^. In addition, peaks observed at 2850 cm^−1^ and 2920 cm^−1^ were related to the symmetric and asymmetric vibrations of C–H bonds of PVA. Finally, peaks observed in the 3600–3300 cm^−1^ range were related to the stretching vibrations of O–H and N–H bonds due to the presence of hydroxyl groups of PVA and amine groups of SF^36,43^. The FTIR spectra of hybrid structure showed characteristic peaks of the both nanofibrous and hydrogel layers. Hydroxyl stretching vibrations of the GT, mannuronic acid/guluronic acid units of SA show absorption bands at 3436 cm^−1^. The peak at 2923.2 cm^−1^ and 2856.6 cm^−1^ are related to C–H stretching vibrations of GT, mannuronic acid/guluronic acid units of SA, while the peaks at 1654.9 cm^−1^ are due to C=O stretching vibrations of –COOH and COO^−^ groups of galactouronic acid of GT and mannuronic acid/guluronic acid units of SA. The peaks at about 1048.6 cm^−1^ are also due to C–O stretching vibrations of pyranose rings of GT, C–O stretching vibrations of mannuronic acid and guluronic acids units of SA^18^.

### Thermal properties of hybrid wound dressing

The thermal properties of prepared hybrid wound dressing were investigated using TGA and results are shown in Fig. 6c. The final char yield of the sample in the air conditions was 45.65%, showing the desired thermal stability of prepared hybrid structure. Initially, the sample demonstrated a small (6%) weight loss due to the evaporation of physically bound water and moisture, followed by a 4% weight loss up to 210 °C. The following endo­therm region at about 220 °C on the TGA curves of the sample was attributed to a physical transition, such as the melting of crystalline phase, and not to a chemical decomposition. The weight loss in the 250 °C region was attributed to GT, and major decomposition occurred up to 307 °C. In general, the decomposition process included desorption of physically-bound water, removal of structural water (dehydration process), depolymerization as well as the rupture of C–O and C–C bonds in the ring units, which led to the formation of CO, CO_2_ and H_2_O, and finally, the formation of graphitic carbon substances (char). In addition, semi-plateau regions could be attributed to the hydrophilic nature of functional groups in polymers^44^.

### The result of mechanical test

The results of tensile analysis on SF/PVA nanofibrous web, SA/GT and SF/PVA-SA/GT hybrid structure are summarized in Table 1. From the results, the mechanical strength of nanofibrous layer is almost weak. It was observed that the incorporation of SA/GT hydrogel in the hybrid matrix considerably enhanced the mechanical properties. Combination of the nanofibrous layer into the hydrogel increased the elongation at break and the value of young’s modulus of the hybrid is significantly higher than that of individual layers. It could be due to high mechanical properties of tragacanth in hydrogel^22^.

**Table 1:** Mechanical properties of SF/PVA nanofibers, SA/GT hydrogel and the hybrid structure.

Sample	Tensile strength (MPa)	Young’s modulus (MPa)	Elongation at break (%)	Work to breaking point (J/cm^3^)
SF/PVA nanofibrous web	0.29 ± 0.01	0.78 ± 0.03	8.42 ± 0.05	0.28 ± 0.02
SA/GT hydrogel	2.96 ± 0.04	4.05 ± 0.05	2.06 ± 0.04	2.84 ± 0.09
SF/PVA-SA/GT hybrid structure	3.15 ± 0.06	4.62 ± 0.07	3.22 ± 0.24	2.91 ± 0.12

### Swelling behaviour of hybrid wound dressing

**Figure 7:**
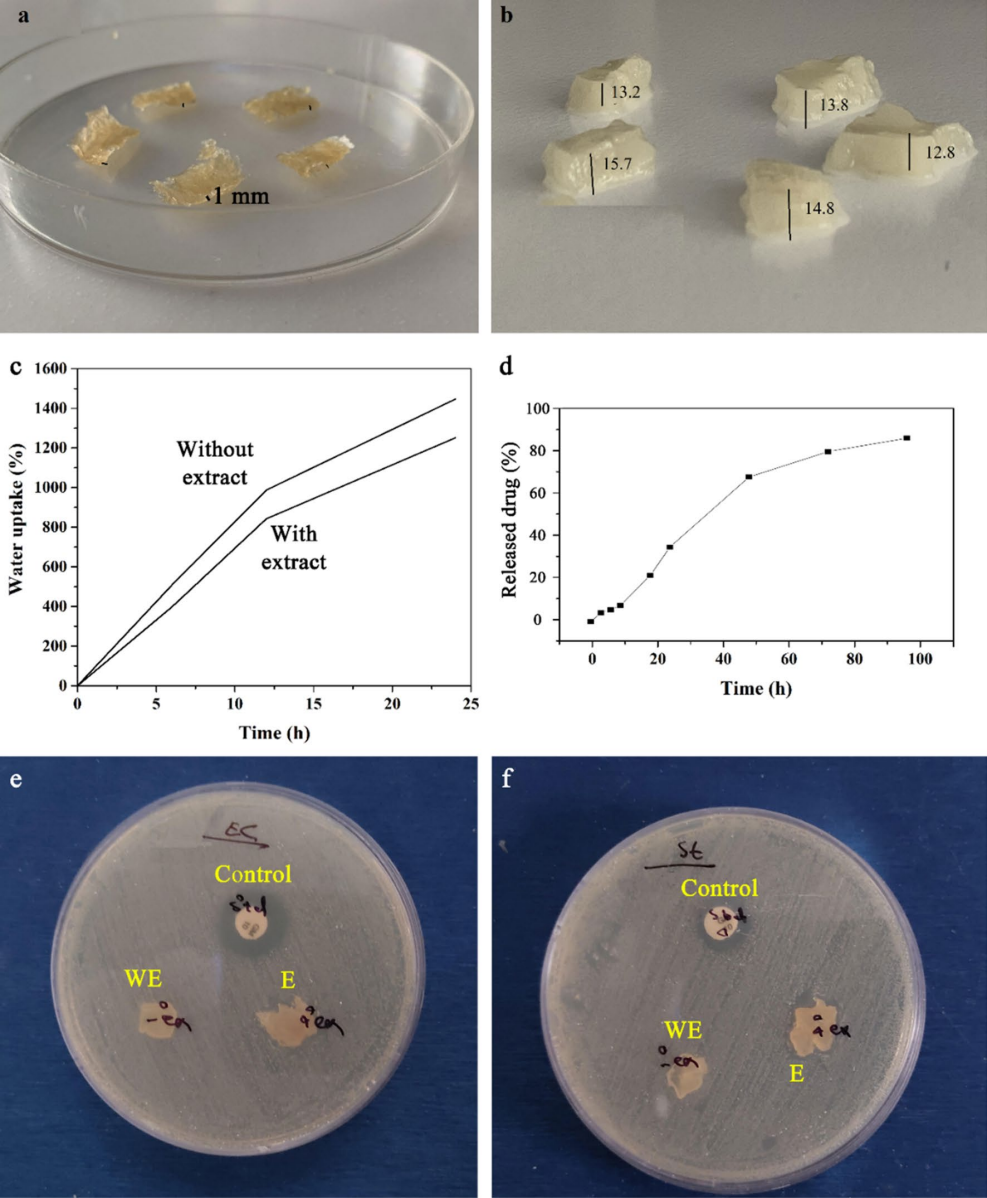
Photographs of hydrogel samples (**a**) before and (**b**) after swelling; (**c**) swelling behavior of hydrogels with and without nanofibers after 24 h; (**d**) drug release behavior from hybrid wound dressing; Inhibition zone against (**e**) *E. coli* and (**f**) *S. Aureus*; in both images control is the antibiotic control disk, WE denotes the hybrid wound dressing without drug, and E denotes the hybrid wound dressing with cardamom oil extract.

Swelling capacity of hydrogels is one of their most important properties when used in biomedical applications such as drug delivery, tissue engineering, and wound healing due to its resemblance with the host tissue. When crosslinked polymer hydrogels are immersed in water or other solvents, they can swell, but they do not dissolve^45^. The swelling property of hydrogels depends on many parameters such as crosslinking density, solvent structure, and polymer–solvent interactions as well as the content of hydrophilic groups in the hydrogel structure. Swelling behaviour of hydrogels also controls biodegradation and sustainable drug release as well^46^. Accordingly, the swelling behaviour of hydrogel and hybrid wound dressing (hydrogel/nanofibers) were investigated and the results were shown in Fig. 7a–c.

As photographically shown in Fig. 7a and b, and according to the obtained diagram (Fig. 7c), hydrogels swelled in distilled water without any changes in their apparent structure after 24 h. The average thickness of samples increased from 1 ± 0.1 to 14 ± 0.1 mm after swelling. Accordingly, 1251% and 1446% swelling values were obtained for the hybrid wound dressing (hydrogel/nanofibers) and hydrogel without nanofibers, respectively. The result concluded that the produced hydrogel can provide a moist environment to accelerate the wound healing process by increasing the rate of mass transfer of biologic substances to the wound site. In addition, hydrogels with favourable swelling properties can absorb the wound exudates, which in turn, prevent infections^47^.

### Drug release studies

In order to investigate the drug release behavior from hybrid wound dressing, UV–Vis spectroscopy was utilized and results are shown in Fig. 7d. Accordingly, calibration curve was first obtained by diluting cardamom oil extract in ethanol and the released content was studied at 0, 3, 6, 9, 18, 24, 48, 72, and 96 h at 240 nm. According to the results, the released content increased over time to 85% release of the drug in total. The release behaviour followed a two-step profile. In the first step (initial 12 h), the drug diffused slowly from the hybrid wound dressing and in the second step (after 12 h), initial burst was observed due to the rapid release from the scaffold, which could be due to the initiation of degradation process of biodegradable constituents. It has been reported that release of pharmaceutical substances from biodegradable polymers can be controlled by the porosity of hydrogel, swelling behaviour, drug loading content and the degradation process as well^48^.

### Antibacterial activity

Antibacterial performance of hybrid hydrogel/nanofiber was investigated against Gram-negative (E. coli) and Gram-positive (*S. Aureus*) using the disk diffusion method (Fig. 7e,f). Results revealed that the hybrid wound dressing (hydrogel/nanofiber) without extract had no antibacterial activity against both bacteria. While for the gentamycin sample as the control, inhibition zones of 17 mm and 16 mm against *E. coli* and *S. Aureus* were observed, this value for the hybrid wound dressing with cardamom oil extract was found to be 14 mm and 12 mm against *E. coli* and *S. Aureus*, respectively. The obtained results are in agreement with the results reported by Najafi et al.^49^, who reported that cardamom extract improved the antibacterial properties of alginate/PEA scaffold.

The activity of sample containing cardamom oil extract was higher against the Gram-positive bacterium (85.35%) compared to the Gram-negative bacterium (75%). Similar results have been observed in other studies, where a higher activity against *S. Aureus* have been observed for cardamom essential oil compared to other bacteria^50^. The activity of cardamom oil extract against bacteria and microorganisms is primarily caused by disturbing the cytoplasmic membrane due to the difference in the surface charges of cell membrane and cyclic hydrocarbons present in the constituents of cardamom, which finally lead to coagulation of cell contents of the bacteria and hence, cell death^49^. It should be mentioned that cardamom contains different bioactive metabolites such as flavonoids, carotenoids, and terpenes which have potential pharmaceutical and clinical properties such as antioxidant, anti-inflammatory, antimicrobial, antivirus, and antibacterial properties^50,51^.

### Cell viability and attachment

**Figure 8:**
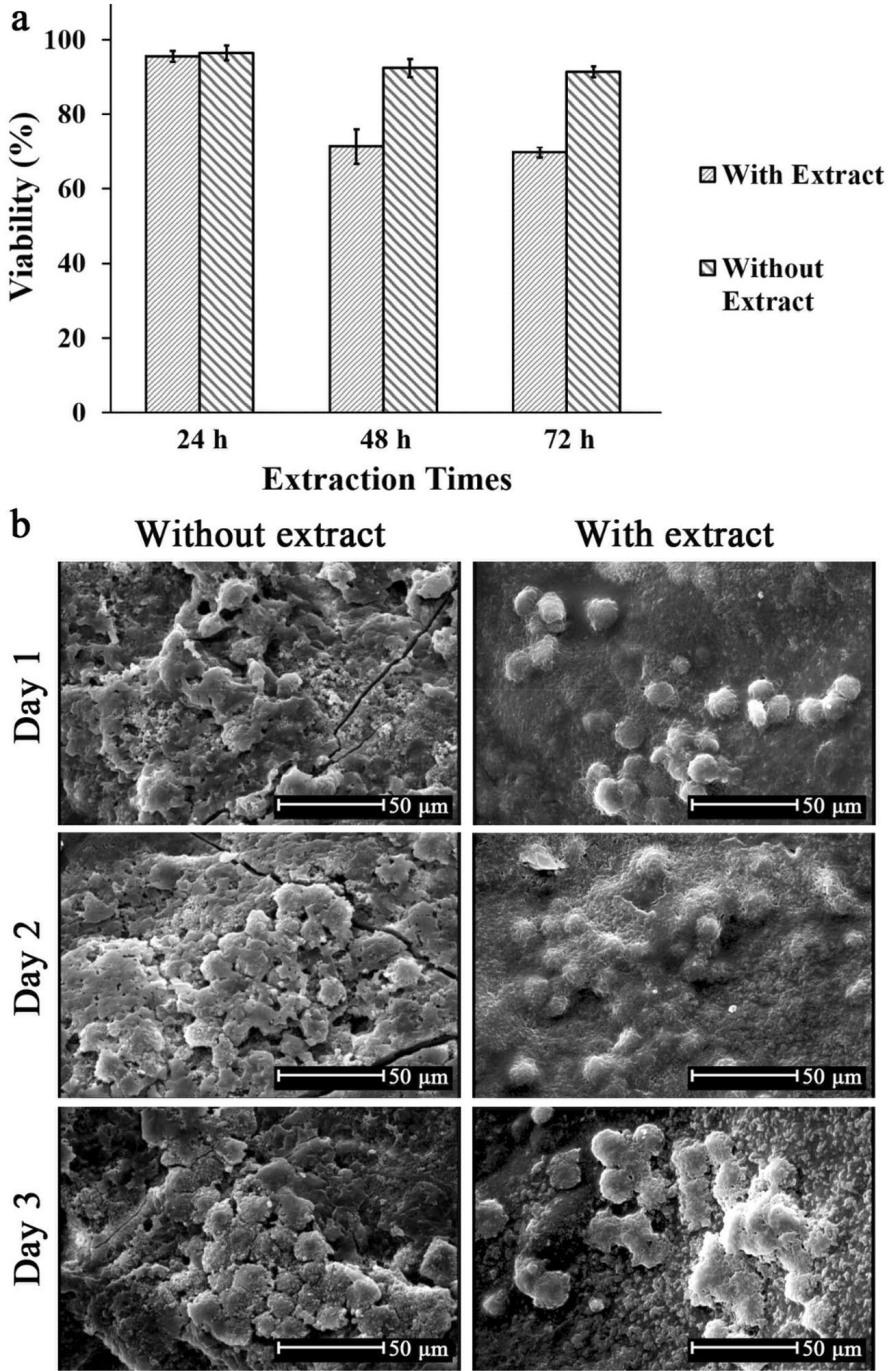
(**a**) MTT assay of controls, hybrid wound dressing without (WE) and with (E) extract after 24, 48, and 72 h (n = 5, the mean difference is significant at the 0.05 level), (**b**) SEM micrographs showing cell attachment behavior on hybrid wound dressing with and without extract after 1, 2, and 3 days of culture.

Cell viability, as an index of biocompatibility, was investigated using fibroblast cells for hybrid wound dressing with (E) and without cardamom oil extract (WE) and results are shown in Fig. 8a. In general, the samples had cell viability in the initial 24 h. However, the sample without extract caused higher cell viability than its counterpart with extract after 48 and 72 h of cell culture. Jamil et al. reported that for systems in which cardamom oil extract was encapsulated in chitosan nanocomposites, the extract reduced cell viability^52^.

Cell attachment studies were also carried out after 1, 2, 3 days using SEM, and results are shown in Fig. 8b. In agreement with MTT studies, the number of alive cells on the surface of samples decreased when cardamom extract was added to the hybrid wound dressing. However, cell flattening and spreading was more evident in sample containing extract, particularly after 48 and 72 h. In addition, attachment studies showed that the surface of the sample was favourable host for cells to grow and proliferate that showed their potential for skin tissue regeneration.

## Conclusions

In this study, SF was first extracted from silk cocoons, and XRD and FTIR evaluations confirmed the extraction of fibroin with desirable chemical structure. The uniform fibers were electrospun with SF/PVA ratio of 1:1 under a voltage of 18 kV and a distance of 15 cm. FTIR analysis for nanofibers showed the presence of SF and PVA peaks in the nanofibrous scaffold. Next, a hydrogel based on GT/SA including cardamom oil extract was successfully prepared and crosslinked by gltaraldehyde (GA). Hybrid wound dressing was prepared by placing the nanofibrous structure on the surface of hydrogel prior to final drying stage. The swelling test showed the ability of the hybrid wound dressing to retain water in its network without the structure changing or collapsing. The swelling ratio of hydrogel with and without fibers was 1251% and 1446%, respectively. Incorporation of SA/GT hydrogel enhanced the mechanical properties of the hybrid structure significantly. The release test showed the slow release of the extract from the hybrid hydrogel in the initial hours, followed by a more rapid release due to the swelling and biodegradation. TGA experiment showed the thermal stability of prepared hybrid wound dressing. The antibacterial test was performed against Gram-positive (*S. aureus*) and Gram-negative (*E. coli*) bacteria, with 85.35% activity against *S. aureus* and 75% activity against *E. coli*, showing the excellent antibacterial properties of resulting hybrid system containing cardamom oil extract. While cell cytotoxicity studies showed the decrease in viability of cells for the sample containing cardamom oil extract, they both fell into the category of biocompatibility. In addition, cell attachment studies showed the adhesion, spreading and proliferation of cells after 3 days of culture in the sample containing the extract. Consequently, the results showed that the engineered hybrid wound dressing developed in this study could be a promising candidate for skin tissue engineering and wound healing applications and can be used for further in vivo and clinical studies.

## Data Availability

All data generated or analyzed during this study are included in this published article.
